# Halofuginone inhibits radiotherapy-induced epithelial-mesenchymal transition in lung cancer

**DOI:** 10.18632/oncotarget.11217

**Published:** 2016-08-11

**Authors:** Yang Chen, Weishuai Liu, Peng Wang, Hailing Hou, Ningbo Liu, Linlin Gong, Youyou Wang, Kai Ji, Lujun Zhao, Ping Wang

**Affiliations:** ^1^ Department of Radiation Oncology, Center of Cancer, Key Laboratory of Cancer Prevention and Therapy, Tianjin 300060, China; ^2^ Department of Pain Management, Tianjin Medical University Cancer Institute and Hospital, National Clinical Research, Center of Cancer, Key Laboratory of Cancer Prevention and Therapy, Tianjin 300060, China; ^3^ Department of Radiation Oncology, Peking University International Hospital, Beijing 102206, China

**Keywords:** epithelial-mesenchymal transition (EMT), halofuginone, transforming growth factor-β (TGF-β1), radiotherapy, lung cancer

## Abstract

Radiotherapy is used to treat many different human tumors. Paradoxically, radiation can activate TGF-β1 signaling and induce the epithelial-mesenchymal transition (EMT), which is associated with enhanced tumor progression. This study investigated the inhibitory effects of halofuginone, a plant-derived alkaloid that has been shown to inhibit TGF-β1 signaling, on radiation-induced EMT and explored the underlying mechanisms using a Lewis lung carcinoma (LLC) xenograft model. The cells and animals were divided into five treatment groups: Normal Control (NC), Halofuginone alone (HF), Radiotherapy alone (RT), Radiotherapy combined with Halofuginone (RT+HF), and Radiotherapy combined with the TGF-β1 inhibitor SB431542 (RT+SB). Radiation induced EMT in lung cancer cells and xenografts, as evidenced by increased expression of the mesenchymal markers N-cadherin and Vimentin, and reduced expression of the epithelial markers E-cadherin and Cytokeratin. Further, radiotherapy treatment increased the migration and invasion of LLC cells. Halofuginone reversed the EMT induced by radiotherapy *in vitro* and *in vivo*, and inhibited the migration and invasion of LLC cells. In addition, TGF-β1/Smad signaling was activated by radiotherapy and the mRNA expression of Twist and Snail was elevated; this effect was reversed by halofuginone or the TGF-β1 inhibitor SB431542. Our results demonstrate that halofuginone inhibits radiation-induced EMT, and suggest that suppression of TGF-β1 signaling may be responsible for this effect.

## INTRODUCTION

Epithelial-mesenchymal transition (EMT) is an essential process during embryonic development that also has a profound impact on cancer progression. In normal and cancer cells EMT can be activated by multiple signaling pathways such as TGF-β1, Wnt–β-catenin, and Notch [1]. Radiotherapy is a valuable tool in the treatment of many human cancers used in combination with chemotherapy and/or surgery, and has been implicated in the induction of a malignant phenotype consistent with EMT in colorectal cancer cells [2]. Radiation increases the expression of TGF-β1 [3], which plays an important role in tumor invasion and metastasis [4], [5]. Recent studies indicate that halofuginone, a plant-derived alkaloid, can inhibit TGF-β1 signaling pathway by multiple mechanisms [6]. Halofuginone blocks TGF-β1 signaling in epithelial cells by inhibiting the phosphorylation and activation of Smad2 and Smad3, and by inducing Smad7 expression [7]. Halofuginone therapy has been shown to reduce the development and progression of bone metastasis caused by melanoma cells by inhibiting TGF-β1 signaling [8]. Our previous studies have demonstrated that the combination of halofuginone and radiotherapy inhibits hepatic and pulmonary metastases [9], but the underlying mechanisms have not been established. EMT is one of the most important factors associated tumor metastasis, to date there have been no studies on the use of halofuginone to prevent tumor cell EMT. In this study we investigated the potential of halofuginone to prevent tumor cell EMT. We report for the first time that halofuginone can block TGF-β1 signaling to suppress radiation induced EMT. Our findings suggest that radiotherapy combined with halofuginone may be a novel treatment for lung cancer.

## RESULTS

### Irradiation induces LLC cell EMT and enhances invasion

**Figure 1:**
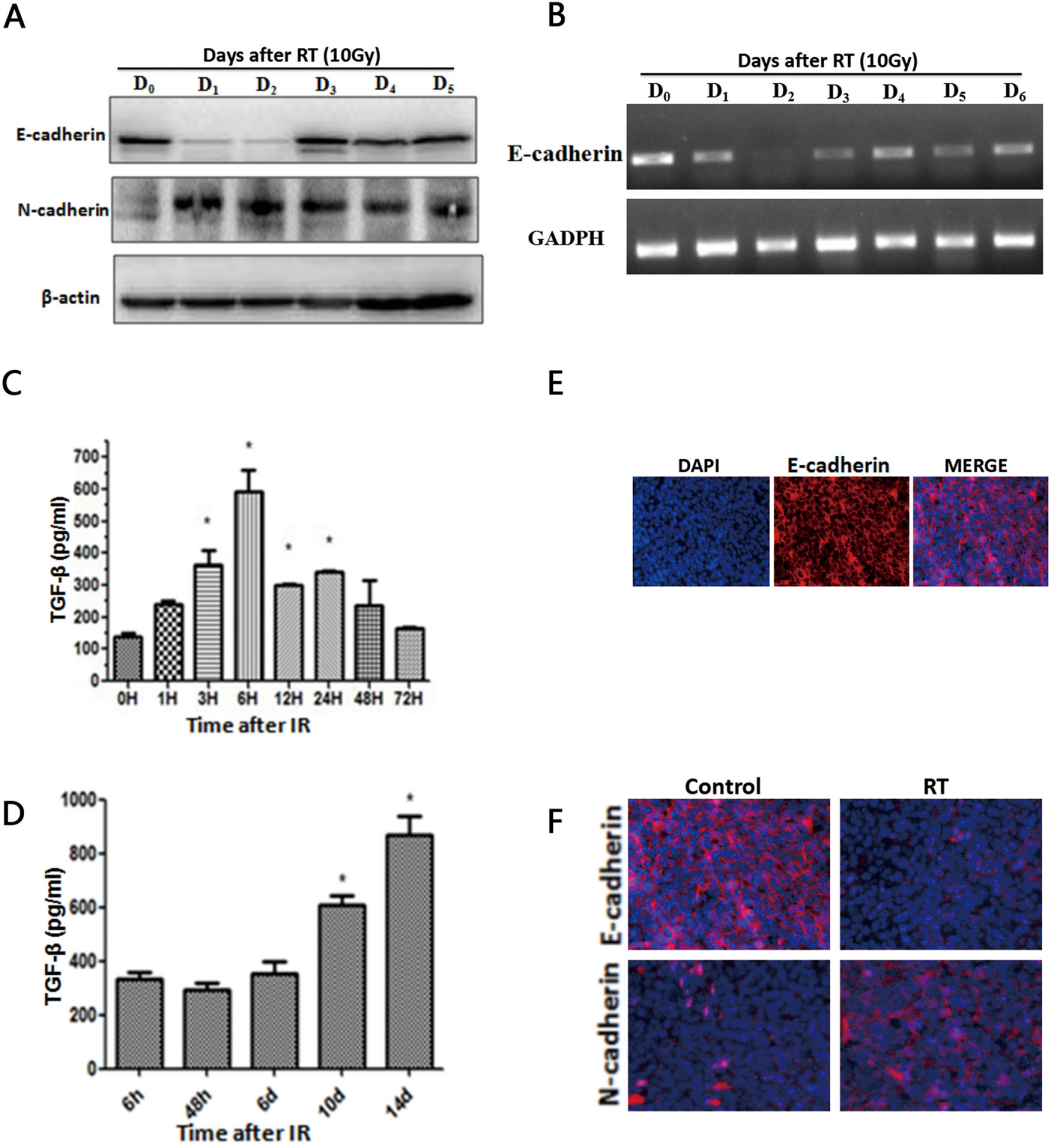
Radiotherapy induces TGF-β1 and an EMT-like phenotype **A.** Western blot of LLC lysates showing E-cadherin and N-cadherin levels after radiotherapy. **B.** mRNA expression of E-cadherin and respective GAPDH. **C.** ELISA assays estimate TGF-β1 levels in LLC cells followed by different times of radiotherapy. **D.** ELISA assays estimate TGF-β1 levels in tumor tissue followed by different times of radiotherapy. Each experiment was repeated at least three times. Error bars indicate standard deviation. * represents level of significance with *P* < 0.05 with respect to control. **E.** Immunofluorescent staining of E-cadherin (red) in control group xenograft tumors tissues 14 days after treatment. Nuclei were stained with DAPI (blue). Merged images show epithelial marker E-cadherin at the cytomembrane. **F.** Immunofluorescence results show that compared to the NC group, in the RT group the expression of epithelial markers E-cadherin was reduced and the expression of the mesenchymal marker N-cadherin was enhanced.

Western blots of LLC lysates showed that expression of the epithelial marker E-Cadherin was decreased at day 1, and 2 and expression of the mesenchymal marker N-Cadherin was enhanced at day 1, and 2 following radiotherapy (Figure 1A). mRNA expression of E-Cadherin was also reduced after irradiation (Figure 1B). ELISA results showed that the level of TGF-β1 in LLC cells was significantly increased 3h, 6h, 12h, and 24h after radiotherapy (*P* < 0.05) (Figure 1C). TGF-β1 levels in tumor tissue were significantly increased 10d and 14d after radiotherapy (*P* < 0.05) (Figure 1D).

**Figure 2:**
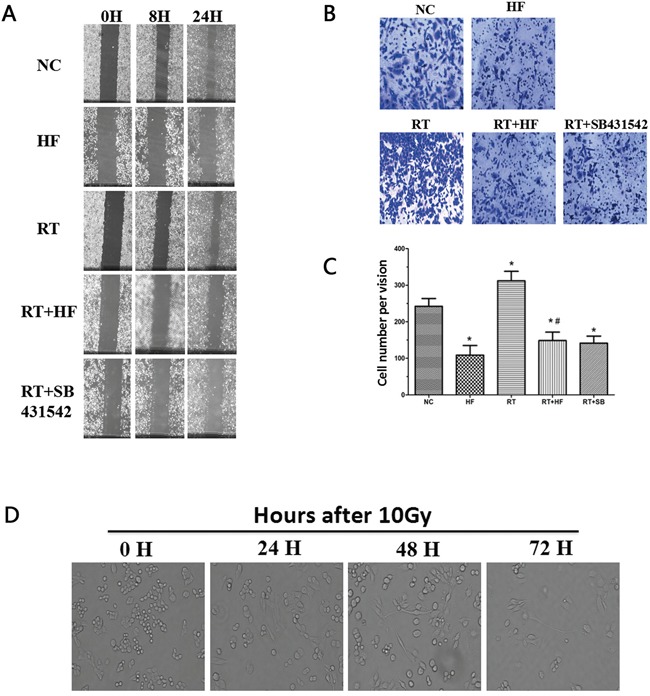
Migration and wound healing assays **A.** Wound healing assay to compare the migratory capabilities of the cells. The images were acquired at ×100 magnification. **B.** Transwell chamber assay. The images were acquired at ×200 magnification. **C.** Cell numbers were counted as an average of eight high-magnification fields. *represents a significant difference (*P* < 0.05) compared to the control group. #represents a significant difference (*P* < 0.05) compared to the irradiation group. **D.** Images of cell morphology after radiotherapy.

Immunofluorescence results show that E-Cadherin is expressed in xenograft tumors tissues without any treatment (Figure 1E), and E-cadherin expression is suppressed in the RT group after irradiation, while expression of the mesenchymal marker N-cadherin is enhanced in the RT group (Figure 1F). The scratch assay and transwell chamber assay indicated that radiotherapy treatment increased the ability of LLC cells to migrate (Figure 2A). Together, these findings indicate that irradiation can induce LLC cell EMT and increase cell migration.

### Halofuginone inhibits migratory ability of LLC cells

EMT is a biological process during which epithelial cells are converted to mesenchymal cells, and EMT can increase the migratory ability of tumor cells. Radiotherapy combined with halofuginone reduced the ability of cells to migrate, suggesting an inhibition of EMT. Irradiation increased TGF-β1 levels, so we used SB431542, a TGF-β1 inhibitor, combined with radiotherapy as a positive control. Wound healing assays (Figure 2A) and cell migration assays (Figure 2B and 2C) indicated that compared with the NC group, scratch wound healing and transwell migration were reduced in the HF group (*P*<0.05). Twenty-four hours after irradiation the scratch wound healing was more pronounced, and the number of the cells transferred to the lower surface in the transwell chamber was increased in RT group (*P* < 0.05) compared to the HF group. The areas of scratch wound healing were reduced and the numbers of the cells transferred to the lower surface in transwell chamber were decreased in RT+HF group and the RT+SB group (*P* < 0.05) compared to the RT group. After 10 Gy radiation treatment, LLC cells developed a spindle-like morphology and a fibroblast-like appearance. These cells showed a distinct morphology with long protrusions and microspikes (Figure 2D). These results suggest that irradiated cells are more aggressive and metastatic.

### Halofuginone may reverse radiotherapy-induced EMT in lung cancer cells

**Figure 3:**
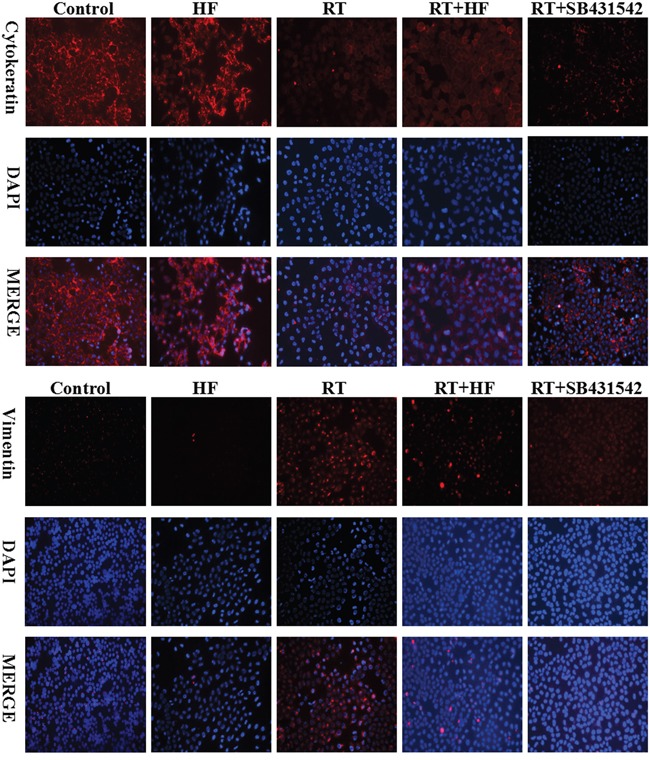
Cytokeratin and vimentin expression in LLC cells Immunofluorescence analysis of cytokeratin (red) and vimentin (red) expression in LLC cells, counterstained with DAPI (blue). Cells were stained 48 hours after indicated treatments.

**Figure 4:**
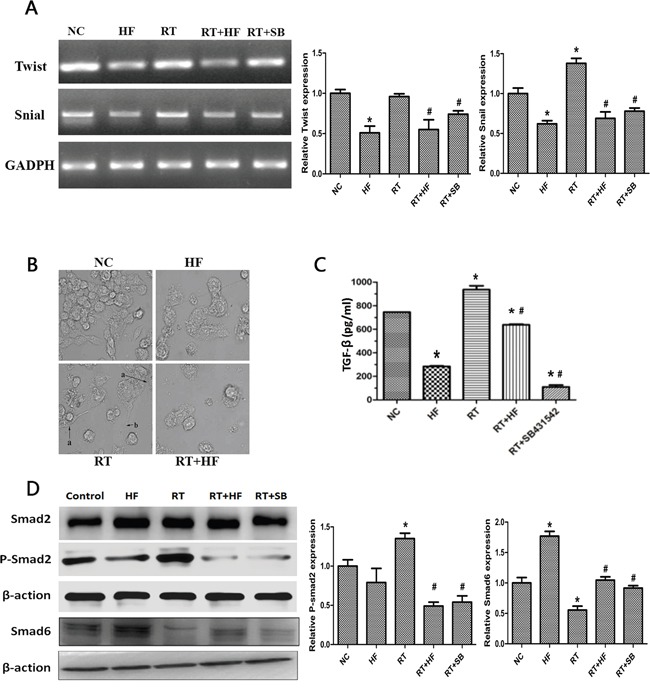
Effects of halofuginone and radiation on TGF-β1 signaling and EMT **A.** mRNA expression of Twist and Snail after 48h of various treatments. **B.** SEM images of cells subjected to various treatment. **C.** Representative histograms of extracellular TGF-β1 levels of various treatments quantitatively determined through ELISA assays. LLC cells were treated with different condition for 48 hours. **D.** Western blot analysis. Cell lysates were collected from LLC cells after indicated treatments, and analyzed for changes in expression of TGF-β1 pathway proteins, Smad2, p-Smad2, and Smad6. Actin served as a loading control. Error Bars, SD. *represents significance level with *P* <0.05 with respect to the NC group. # represents the level of significance with *P* <0.05 with respect to the RT group.

To further confirm the role of halofuginone in inhibiting radiation-induced migration and invasion, we assessed the expression of epithelial markers like E-cadherin and mesenchymal markers like vimentin using immunofluorescence. Expression of the epithelial marker cytokeratin was reduced in the RT group compared to the NC group, but the expression of cytokeratin was enhanced in the HF group compared to the NC group. Furthermore, the expression of cytokeratin in the RT+HF group and RT+SB group was enhanced compared to the RT group; the expression of mesenchymal markers vimentin was enhanced in the RT group compared to the NC group, but its expression was reduced in the HF group compared to the NC group, and its expression in the RT+HF group and RT+SB group was reduced compared to the RT group (Figure 3). Cell morphology in various treatment groups after 24h incubation showed that control group cells exhibited a well spread and compact morphology, whereas the radiotherapy group cells lose cell-cell contacts and acquire migratory properties. However, radiotherapy combined with halofuginone restored the compact morphology (Figure 4B). Twist has been shown to play a major role in cancer progression by promoting EMT and helping the metastatic process [10]. Several groups have reported that high Twist and Snail expression downregulate *E-cadherin and* promote EMT [11]. In this study, the RT-PCR results indicate that the RT group has higher expression of Snail than the NC group, but the expression of Twist and Snail are reduced in the HF group. Although Twist expression is highest in the NC group, the RT+HF and RT+SB groups exhibited reduced expression of Twist and Snail compared with the RT group (Figure 4A).

### Combination therapy with halofuginone and radiation inhibits tumor growth and reverses EMT

**Figure 5:**
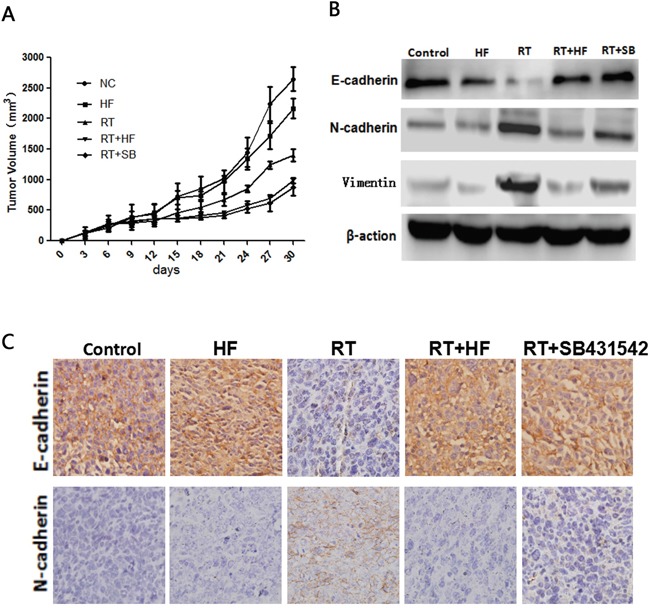
Radiotherapy combined with halofuginone restrains tumor growth **A.** Tumor growth assay. Tumors were measured twice weekly, and tumor volumes were determined from caliper measurements of tumor length (L) and width (W) according to the formula (L × W^2^)/2. The growth curves represent the average values of 6 mice in each group. Error bars indicate standard deviation. **B.** Western blot. Proteins were collected from tumor tissues after 14 days of indicated treatments and analyzed for changes in expression of EMT markers, E-cadherin, N-cadherin and vimentin. Actin served as a loading control. **C.** IHC staining. Xenograft tumors tissues were collected after 14 days of indicated treatments. Immunostaining was performed to test the changes of E-cadherin and N-cadherin.

We further extended our study to an *in vivo* model. LLC cells were inoculated into female C57BL/6J mice (6 weeks old, 16-18 g) to establish xenografts. Our results showed that combination treatment with radiotherapy and halofuginone significantly inhibited tumor growth compared to halofuginone or radiotherapy alone (Figure 5A). Immunohistochemistry results showed that in the RT+HF group and RT+SB group, the expression of the epithelial marker E-cadherin was enhanced and expression of the mesenchymal marker N-cadherin was reduced compared to the RT group. The expression of E-cadherin in the RT group was reduced compared to the NC and HF groups, but the expression of E-cadherin in the RT+HF group and RT+SB group was elevated compared to the RT group. The expression of N-cadherin in the RT group was elevated compared to the NC and HF groups, but its expression in the RT+HF group and RT+SB group was reduced compared to the RT group (Figure 5C). Our findings show that combination treatment with radiotherapy and halofuginone can reverse radiotherapy-induced EMT in xenograft tumors. Western Blot studies revealed reduced levels of E-cadherin and elevated levels of N-cadherin and Vimentin in tumors treated with radiation alone, whereas these effects were reverse in the RT+HF group and RT+SB group (Figure 5B).

### TGF-β1/Smad signaling was activated by radiotherapy and this effect was reversed by halofuginone

**Figure 6:**
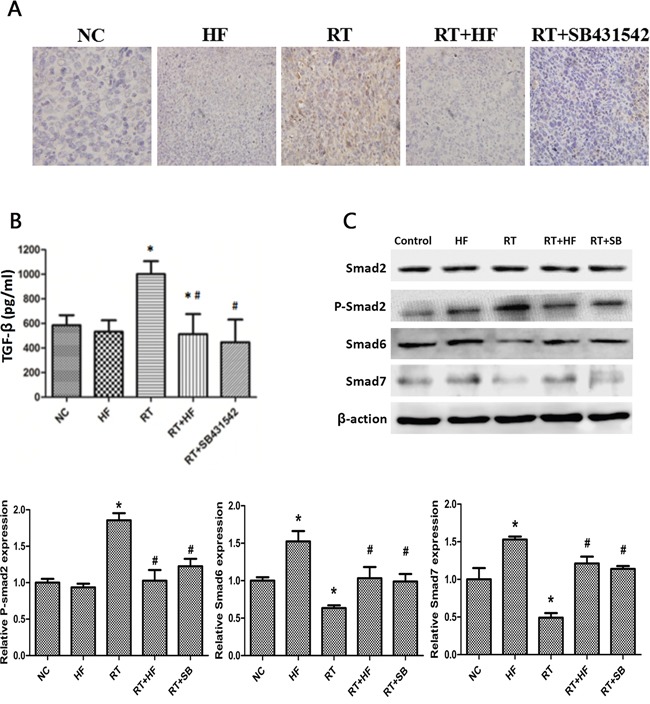
Effects of halofuginone on TGF-β1 signaling in LLC xenografts **A.** IHC staining: xenograft tumors tissues were collected after 14 days of indicated treatments. The expression of TGF-β1 was located in the cytoplasm or stroma, and after the IHC process, positive area turned into tan color. **B.** ELISA assays measure TGF-β1 levels in the tumor tissue after various treatments. Tumors tissues were collected after 14 days of treatment. **C.** Western blot analysis. Proteins were collected from tumor tissues after indicated treatments, and analyzed for changes in expression of TGF-β1 signal pathway markers, Smad2, p-Smad2, and Smad6 proteins. Actin served as a loading control. Tumor tissues lysates were collected from xenograft tumors tissues after 14 days of indicated treatments. Error Bars, SD. *represents significance level with *P* < 0.05 with respect to the NC group. # represents the level of significance with *P* < 0.05 with respect to the RT group.

To further validate the role of halofuginone in inhibiting radiation-induced EMT, we assessed the expression of TGF-β1 and its downstream signaling proteins. First, extracellular TGF-β1 was quantified by ELISA assay. ELISA results showed that the level of TGF-β1 in the HF group was lower than in the NC group (*P* < 0.05), but the level of TGF-β1 in the RT group was higher than in the NC group (*P* < 0.05). The level of TGF-β1 in the RT+HF group and RT+SB group was significantly decreased compared to the RT group (*P* < 0.05) (Figure 4C). Quantification of xenograft TGF-β1 levels by ELISA assay revealed that the levels of TGF-β1 in the RT group was higher than in the NC group (*P* < 0.05), but the level of TGF-β1 in the RT+HF group and RT+SB group was significantly lower than in the RT group (*P* < 0.05) (Figure 6B). Also, immunohistochemistry showed that the expression of TGF-β1 in the RT group was increased compared to the control group, and the expression of TGF-β1 was lower in the RT+HF group and RT+SB group (Figure 6A). The Smad proteins are a family of transcription factors found in vertebrates, insects and nematodes [12]. Smad2 and Smad3 were recognized as receptor-activated Smads (R-Smads) [13]. Smad4 is a common-partner Smad (Co-Smad) of TGF-β1 signaling which translocates efficiently to the nucleus [14]. I-Smads include Smad6 and Smad7, which can prevent R-Smads from accessing the activated type I receptor [15]. SMAD6 can inhibit BMP signaling, and Smad7 acts as a general inhibitor of TGF-β1 family signaling [16]. SMAD2 and SMAD3 recruit the common-mediator SMAD, SMAD4, forming a heteromeric complex. The complex translocates to the nucleus where it regulates the transcription of target genes. Under normal physiological conditions, I-SMADs are involved in maintaining cellular homeostasis. However, in cancer, I-SMADs can block the activation of R-SMADs and co-SMADs, and the overexpression of I-SMADs is positively correlated with multiple tumor types including breast, colorectal, and prostate cancers [17]. Western Blots showed that TGF-β1/Smad signaling both in LLC cells (Figure 4D) and xenografts (Figure 6C) was activated by radiotherapy, and this effect was reversed by halofuginone or the TGF-β1 inhibitor SB431542. p-Smad2 was increased in the RT group and reduced in the two combination groups. The expression of Smad6 and Smad7 in the RT group was reduced compared to the NC group, whereas these proteins were significantly restored in the RT+HF group and the RT+SB group.

## DISCUSSION

Our results indicate that halofuginone is a promising therapeutic agent for the inhibition of radiation therapy–induced EMT, and this effect may be mediated by inhibition of TGF-β1 signaling. EMT is a fundamental biological process during embryonic development during which epithelial cells convert to mesenchymal cells [18]. EMT is a complex program accompanied by the loss of epithelial markers such as adherens proteins E-cadherin and the acquisition of mesenchymal markers such as N-cadherin and vimentin. Irradiated cells have been shown to acquire a more mesenchymal-like morphology [19]. EMT allows tumor cells become more invasive, survive in the circulation, and form a distant metastasis [20]. Our early studies have shown that halofuginone can both significantly downregulate type I collagen and microvessel density counts, and suppress the development of hepatic and pulmonary metastases [9]. Here we found a similar response in LLC cells and xenografts with reduced E-cadherin levels and upregulation of N-cadherin after irradiation. These results are similar to the mRNA levels of E-cadherin confirmed in RT-PCR studies.

TGF-β1 signaling regulates several different biological processes involving cell-growth, differentiation, apoptosis, motility, angiogenesis, and EMT [21]. TGF-β1 signaling plays complex roles during tumor progression and can either inhibit or promote tumor growth depending on the cellular context [22]. The role of TGF-β1 in inducing EMT is complex too. TGF-β1 can induce EMT via Smad-dependent signaling, that is, via Smad2 and Smad3. TGF-β1–induces Smad2 phosphorylation, and p-smad2 multimerizes with co-mediator Smad4 to translocate into the nucleus to regulate the transcription of target genes [23]. In addition, TGF-β1 can induce EMT via a Smad-independent pathway. TGF-β1 can promote H-Ras-mediated cell migration and invasion in which p38 MAPK and ERK signaling pathways are involved [24]. In addition to the MAPK signaling pathway, *in vivo* experiments have established that inhibition of NF-κB signaling prevents EMT in epithelial cells, while activation of this pathway promotes the transition to a mesenchymal phenotype even in the absence of TGF-β1 [25]. Interestingly, recent studies have demonstrated that phospho-Twist1 mediates cross-talk between PI3K/Akt and TGF-β1 signaling and promotes tumor metastasis [26]. It has been suggested that mTORC1 inhibition can suppress the TGF-β1 induced translation pathway and increase cell size without affecting the EMT phenotype. Additionally, decreases in the migratory and invasive behavior of cells accompany TGF-β1 induced EMT [27]. Other results suggest that the mTORC2 pathway is an essential downstream component of TGF-β1 signaling, and represents a responsive target to inhibit EMT and prevent cancer cell invasion and metastasis [28].

In our study the ELISA results revealed that radiotherapy can increase the level of TGF-β1. After irradiation the expression of epithelial markers was reduced and mesenchymal markers were enhanced, and this effect was reversed by halofuginone. Our results suggest that halofuginone combined with radiotherapy may prevent radiation-induced increases in TGF-β1. Further, halofuginone reversed radiation induced EMT.

A number of studies have reported that EMT-inducing transcription factors (EMT-TFs), such as Twist, Snail, Slug, and Zeb, are directly or indirectly involved in cancer cell metastasis [29]. Many transcriptional repressors including Snail and Twist play important role in role in TGF-β1 induced EMT [30]. Further we assessed the levels of Snail and Twist in RT-PCR studies, which were significantly elevated after irradiation and this effect was reversed by halofuginone.

One recent study demonstrated that halofuginone could reduce tumor growth [31]. However, in our *in vivo* study halofuginone alone cannot significantly reduce tumor growth, but combination treatment with radiation and halofuginone caused significant inhibition of tumor growth compared to halofuginone or radiation alone. Previous studies have demonstrated that halofuginone can inhibit the activation of fibroblasts and the phosphorylation of Smad3 [32]. Our results indicate that halofuginone can block the TGF-β1/Smad pathway and inhibit radiotherapy-induced EMT.

We conclude that the combination of halofuginone and radiation therapy inhibits EMT in lung cancer cells and xenografts induced by irradiation. This may be due to the inhibition of TGF-β1 signaling. In summary, halofuginone therapy reduced EMT in mice, demonstrating its potential as a therapeutic agent. Radiotherapy combined with halofuginone is a novel treatment for metastases that could be brought to the clinic for the prevention of EMT.

## MATERIALS AND METHODS

### Cell lines

LLC cells were provided by the Tianjin Medical University Cancer Institute and Hospital. These cells were cultured in high glucose DMEM (Gibco Company, USA) supplemented with 10% fetal bovine serum (FBS), 100 U/ml penicillin and 100 U/ml streptomycin and maintained at 37°C in a humidified atmosphere with 5% CO_2_. Cells were cultured overnight to reach 50–70% confluence and divided into five treatment groups: Normal Control (NC), receiving no treatment; Halofuginone alone (HF), maintained in cell culture media containing 58 ng/ml halofuginone; Radiotherapy alone (RT), receiving 6MV X-ray irradiation with 10 Gy/f×1f; Radiotherapy combined with halofuginone (RT+HF), maintained in cell culture media containing 58 ng/ml halofuginone for 24 h, followed by ionizing radiation at a dose of 10Gy/f×1f ; and Radiotherapy combined with TGF-β1 inhibitor SB431542 treatment (RT+SB), maintained in cell culture media containing 40 μg/ml SB431542 for 24 h, followed by irradiation at a dose of 10Gy/f×1f.

### Wound healing assay

Wound healing assays were performed in the NC, HF, RT, RT+HF, and RT+SB cells. The cells were plated at a density of 2×10^5^ cells/well, cultured overnight, and treated as indicated (HF, RT, RT+HF or RT+SB). A scratch wound was created 12h after treatment to wound the monolayer cultured cells in a 6-well plate using a sterile pipette tip, and photomicrographs of the initial wounds were taken. For each condition, three scratches were inflicted in three independent wells of a 6-well plate. Images of the wound closure were taken at 0h, 12h, and 32h after scratching using a digital camera mounted on an inverted microscope. The wound healing was measured using Image-J image analysis software.

### Cell migration and invasion assays

Cell migration through 8.0 μm pore polycarbonate filter inserts in 24-well plates was measured in the NC, HF, RT, RT+HF, and RT+SB cells. After 24h treatment, 5 × 10^4^ LLC cells were added into the upper chamber in serum-free DMEM, and DMEM containing 10% FBS was added to the bottom chamber. The cells were incubated for 48 h, fixed in 4% paraformaldehyde, and stained with 0.1% crystal violet for 30 min. The non-migrated cells were removed from the upper surface of the membrane with a cotton swab. Cells that migrated from the upper chamber to the bottom chamber were counted under 200 × magnification, using the mean count from 10 random fields of view.

### Western blot analysis

For *in vitro* studies, NC, HF, RT, RT+HF, and RT+SB cells were treated as described above, collected 48 hours after irradiation, washed with cold PBS, and lysed in RIPA buffer. For *in vivo* experiments, tumor tissues were collected from the tumor xenografts after 14 days of the indicated treatment. The tumor tissues were homogenized and lysed in RIPA buffer. Protein concentrations were determined using Bradford Reagent (Bio-Rad Laboratories) by measuring absorbance at 562 nm. The proteins lysates were separated using SDS-PAGE (8-12%) and transferred to a polyvinylidene difluoride membrane. The membrane was blocked in 5% nonfat dry milk in TBST for 2 hours at room temperature and then incubated overnight at 4°C with primary antibodies against E-cadherin (#3195, 1:1000, CST), N-cadherin (#4061, 1:1000, CST), Vimentin (#5741, 1:1000, CST), and β-action(#3700, 1:1000, CST). The next day, the membrane was incubated with horseradish peroxidase-conjugated goat anti-rabbit secondary antibody (1:2000 dilution; Cell Signal Technology) for 1 hour at room temperature. Band intensity was analyzed using Quantity One 1 image analysis software (Bio-Rad). The bands were quantitatively analyzed by comparing the expression levels to β-action controls.

### Reverse transcription PCR

Reverse transcriptase-PCR assay was performed to measure the expression of Twist and Snail mRNA. Total RNA was extracted from the lysates of LLC cells treated with nothing, HF, RT, RT+HF, and RT+SB 48h after irradiation. cDNA was synthesized from RNA using the Masterscript RT-PCR System according to the manufacturer's instructions, and stored at −20°C until the assay was performed. cDNA was subjected to PCR with primers for Twist (forward, 5′-TCCACAAGCACCAAGAGTC-3′; reverse, 5′-TGA GGGAGGTAGGGAAGTG-3′), Snail (forward, 5′-GC CACCTTCTTTGAGGTACAAC-3′; reverse, 5′-ATTATT CATGGTCCCTTCTGAG-3′), E-cadherin (forward, 5′-CG GAGGAGAGCGGTGGTCAAAG-3′; reverse, 5′-CTA GTCGTCCTCGCCGCCTCC-3′), and GAPDH (forward, 5′-GTTCGACAGTCAGCCGCATCT-3′; reverse, 5′-CCT GCAAATGAGCCCCAGCCT-3′). PCR was performed in an automatic thermocycler for 40 cycles of: 94°C for 30s, 55°C for 30s, and 72°C for 40s. Gene expression was quantified using Image J software. GAPDH was used as an internal control.

### Immunofluorescence

LLC cells at a density of 1×10^5^ cells/well were cultured on coverslips in 6-well plates. After 24h of incubation, the NC, HF, RT, RT+HF, and RT+SB cells were treated as indicated, and the cells were further incubated for 48h. Cells were fixed in 4% paraformaldehyde for 15 min at 4°C, permeabilized with 1% Triton-100 for 30 min at room temperature. Coverslips were incubated in primary antibodies against Cytokeratin (#9384, 1:1000, CST) or Vimentin (#5741, 1:1000, CST) overnight at 4°C, followed by Alexa fluor 488-conjugated goat anti-rabbit IgG (H + L) secondary antibody (Invitrogen) for 1h at 37°C. Cells were washed and nuclei were counterstained with 1 mg/ml DAPI. Images were acquired with an OLYMPUS BX61 confocal microscope with a 40×objective. The results from at least 3 different experiments were averaged.

### *In vivo* tumor growth assay

Female C57BL/6J mice (6 weeks old, 16–18 g) were used for xenograft experiments. LLC cells (1×10^6^ in 0.2 ml normal saline) were injected subcutaneously into the right thigh of mice. When the average tumor volume reached 200 mm^3^, the mice were randomized into 5 groups to receive the following treatments: Control group (NC); HF (30 μg/ml per day, days 1-14) group; RT (10Gy/f×2f on day 3 and 4) group; RT (10Gy/f×2f on day 3 and 4)+HF (30 μg/d per day, days 14-) group; RT (10Gy/f×2f on day 3 and 4) +(1 μmol/L) SB (1 μmol/L, 0.1 ml/day days 1-14) group. Tumors were measured three times per week, and tumor volumes were calculated using the formula: volume = width ×width× length×0.5. All the procedures are in accord with the guidelines of the laboratory animal ethics committee of Tianjin Medical University.

### Immunohistochemistry

LLC xenograft tumors were collected after 14 days of daily treatments. Immunostaining was performed on 4-μm-thick sections of formalin-fixed, paraffin-embedded samples. The sections were dewaxed using xylene, dehydrated with gradient ethanol, and rehydrated with PBS. Tissue slides were immersed in 3% hydrogen peroxide for 20 min, followed by incubation in 5% BSA for 30 minutes. Then tissue slides were incubated with anti-TGF-β1 antibody (ab64715, 1:150, Abcam), anti-E-cadherin antibody(# 3195, 1:400, CST), or anti-N-cadherin antibody (ab12221, 1:200, Abcam) at 4°C overnight. After washing with PBS, sections were incubated with a secondary antibody (PV-9000 ZSGB-BIO, China) for 30 min at 37°C. DAB was added to the sections and followed by counter staining with hematoxylin. The sections were air-dried, dehydrated, and observed under a microscope. Staining intensity was scored according to the following criteria: 0 for negative staining, 1 for weak staining, 2 for intermediate staining, and 3 for strong staining. The proportion of cells with positive staining was scored as 1 for 0–25%, 2 for 25–50%, 3 for 50–75%, and 4 for 75–100%. The final staining score was the product of the intensity and proportion scores. A final score ≥ 6 was defined as high expression and a score < 6 was defined as low expression.

### ELISA analysis

ELISA Kits (R&D System) were used to measure TGF-β1 levels in LLC cells and tumor tissues in the treatment groups. Conditioned medium containing TGF-β1 from the treatment groups was diluted in blocking buffer to equalize the protein concentration and incubated in ELISA plates for 2h at 37°C. After washing with PBS three times for 3 minutes, the cells were allowed to react with horseradish peroxidase-labeled secondary antibody for 30 minutes at 37°C. The chromogen tetramethylbenzidine was added to each well and incubated for 30 minutes at 37°C to allow the enzymatic reaction to occur, and the cells were washed with PBS three times for 3 minutes. Finally, the reactions were stopped with the stop solution. The absorbances were measured at 450 nm using a microplate reader. Three independent experiments were performed for each point.

### Statistical analysis

Statistical analyses were performed by using Statistical Product and Service Solutions 18.0 soft-ware package (IBM Corporation, Armonk, NY, USA). Student's t-tests were used to determine the significance between control and treated-groups, and *P*< 0.05 value was considered significant.
