# Correction: Halofuginone inhibits radiotherapy-induced epithelial-mesenchymal transition in lung cancer

**DOI:** 10.18632/oncotarget.25958

**Published:** 2018-08-03

**Authors:** Yang Chen, Weishuai Liu, Peng Wang, Hailing Hou, Ningbo Liu, Linlin Gong, Youyou Wang, Kai Ji, Lujun Zhao, Ping Wang

**Affiliations:** ^1^ Department of Radiation Oncology, Tianjin Medical University Cancer Institute and Hospital, National Clinical Research Center of Cancer, Key Laboratory of Cancer Prevention and Therapy, Tianjin 300060, China; ^2^ Department of Pain Management, Tianjin Medical University Cancer Institute and Hospital, National Clinical Research, Center of Cancer, Key Laboratory of Cancer Prevention and Therapy, Tianjin 300060, China; ^3^ Department of Radiation Oncology, Peking University International Hospital, Beijing 102206, China

**This article has been corrected:** The correct author affiliation is given below:

^1^ Department of Radiation Oncology, Tianjin Medical University Cancer Institute and Hospital, National Clinical Research Center of Cancer, Key Laboratory of Cancer Prevention and Therapy, Tianjin 300060, China

Original article: Oncotarget. 2016; 7:71341-71352. https://doi.org/10.18632/oncotarget.11217

